# Calmodulin Is the Fundamental Regulator of NADK-Mediated NAD Signaling in Plants

**DOI:** 10.3389/fpls.2019.00681

**Published:** 2019-06-05

**Authors:** Li Tai, Bin-Bin Li, Xiu-Min Nie, Peng-Peng Zhang, Chun-Hong Hu, Lu Zhang, Wen-Ting Liu, Wen-Qiang Li, Kun-Ming Chen

**Affiliations:** ^1^State Key Laboratory of Crop Stress Biology in Arid Area/College of Life Sciences, Northwest A&F University, Yangling, China; ^2^Department of General Biology, College of Life Science and Agronomy, Zhoukou Normal University, Zhoukou, China

**Keywords:** calmodulin, NAD kinase, NAD signaling, regulatory mechanism, plants

## Abstract

Calcium (Ca^2+^) signaling and nicotinamide adenine dinucleotide (NAD) signaling are two basic signal regulation pathways in organisms, playing crucial roles in signal transduction, energy metabolism, stress tolerance, and various developmental processes. Notably, calmodulins (CaMs) and NAD kinases (NADKs) are important hubs for connecting these two types of signaling networks, where CaMs are the unique activators of NADKs. NADK is a key enzyme for NADP (including NADP^+^ and NADPH) biosynthesis by phosphorylating NAD (including NAD^+^ and NADH) and therefore, maintains the balance between NAD pool and NADP pool through an allosteric regulation mode. In addition, the two respective derivatives from NAD^+^ (substrate of NADK) and NADP^+^ (product of NADK), cyclic ADP-ribose (cADPR) and nicotinic acid adenine dinucleotide phosphate (NAADP), have been considered to be the important messengers for intracellular Ca^2+^ homeostasis which could finally influence the combination between CaM and NADK, forming a feedback regulation mechanism. In this review article, we briefly summarized the major research advances related to the feedback regulation pathway, which is activated by the interaction of CaM and NADK during plant development and signaling. The theories and fact will lay a solid foundation for further studies related to CaM and NADK and their regulatory mechanisms as well as the NADK-mediated NAD signaling behavior in plant development and response to stress.

## Introduction

The pyridine nucleotide NAD and its phosphorylated relative, NADP, are essential small molecules in living organisms. Many evidence have clearly proved that NAD(P) and their reduced forms NAD(P)H are crucial coenzymes for dehydrogenases (Chen et al., 2016; Grant, 2018; Lin et al., 2018) and vital regulators of anabolic reactions, and thus play important roles in diverse biological processes in most organisms (Pollak et al., 2007). The NAD(H) and NADP(H) are essential molecules with a pronounce activity under oxidative stress (Kirsch and De Groot, 2001). Furthermore, the derivatives of NAD^+^ and NADP^+^ are important messenger molecules in the cytosolic Ca^2+^ stores (Yamasaki et al., 2005; Liu et al., 2009; Meimoun et al., 2009; Gul et al., 2016; Lin et al., 2017). Recently, an extracellular NAD-binding receptor was identified in *Arabidopsis*, indicating that NAD^+^ can act as an endogenous signaling molecule, making NAD signaling an important issue to address (Ziegler, 2000; Wang C. et al., 2017).

NAD kinase (NADK) is the only known enzyme which phosphorylates NAD to synthesize NADP, and maintains the balance between NAD pool and NADP pool in animals, plants and microorganisms through an allosteric regulation mode (McGuinness and Butler, 1985; Kawai et al., 2001a,b; Lerner et al., 2001; Li B.B. et al., 2018). In addition, NADPase, also named NADP phosphatase, dephosphorylates NADP and coordinates the ratio of NADP(H)/NAD(H) with NADK (Richter, 1987; Bhattacharyya et al., 2016). Besides the two enzymes, NADPH oxidases (NOXs), also well-known as respiratory burst oxidase homologs (RBOHs), are also closely related to the redox balance in the organisms (Gupta et al., 2017; Li et al., 2017). NOXs are a class of membrane-localized proteins that oxidize NADPH and release reactive oxygen species (ROS) to the apoplastic spaces of plants, and therefore play crucial roles in stress response and cellular redox homeostasis maintenance (Bedard and Krause, 2007; Cruz-Magalhaes et al., 2018; Hu et al., 2018; Li B.B. et al., 2018). The functions of NOXs are closely associated with NADKs in plant cells, both intracellular redox and ROS homeostasis are relied on the functions of NADK (Li B.B. et al., 2018).

A number of previous studies have shown that calmodulin (CaM), a ubiquitous Ca^2+^ sensor containing four Ca^2+^ binding sites, play important roles in the activation of NADK (Anderson and Cormier, 1978; Anderson et al., 1980; Jarrett et al., 1980; Epel et al., 1981; Simon et al., 1982; Dieter and Marme, 1984; Williams and Jones, 1985). However, it was also found that NADK can be activated indirectly by CaM (Love et al., 2015). Experimental results from the flagellate *Euglena* indicated that NAD^+^, Ca^2+^, CaM, NADK, and NADPase represent the clock “gear” that might form a self-sustained circadian oscillating loop for cell growth (Goto et al., 1985; Laval-Martin et al., 1990). In addition, it was found that a complicated feedback regulation mechanism may exist between CaM and NADK activation. In this regulatory mode, two NAD derivatives, namely cADPR and NAADP, which are generated from NAD^+^ and NADP^+^ respectively, may play key roles. These two NAD derivatives are key regulators of intracellular Ca^2+^ stores (Cancela et al., 1999; Navazio et al., 2000; Patel et al., 2001; Churchill et al., 2002; Berridge et al., 2003; Rutter, 2003; Lee, 2012). As the 2-s messengers of Ca^2+^ signaling, they can affect the binding of Ca^2+^ to CaM by producing intracellular Ca^2+^ transients, and finally, affect the binding of Ca^2+^/CaM complex to NADK. However, although it has been long known that CaM is the key regulator of NADK activation and CaM plays crucial roles in NADK-participated plant stress response and development, the functional mechanism of CaM in NADK activation and NAD signaling is largely unknown yet. In this review, we focus on the recent research advances related to the feedback regulation of the activation of CaM to NADK, hoping to draw out a clear picture of the roles of CaM in NADK-mediated NAD signaling in plant development and stress response.

## Functional Specificity of NAD(H) and NADP(H)

After a long term of evolution, living body cells have established a complete control system that precisely regulates energy metabolism and diverse biological processes during the growth and development of organisms. Each regulatory system requires intermolecular interactions. These molecules will exert activation, inhibition or any other functions after they combined with each other to transmit a variety of external information into the cell. With the advancement of science and technology, research at the molecular level has been increasing in recent decades. Many small molecules’ functions and regulatory mechanisms have been widely explored. For instance, ATP is a direct energy source for cells and its derivative cAMP plays vital roles in many cellular signaling pathways (Huang et al., 2013; Maiellaro et al., 2016; Kato et al., 2017; Wang X. et al., 2017). Coenzyme I (NAD^+^/NADH) and coenzyme II (NADP^+^/NADPH) are also important small signaling molecules which have three major roles: firstly, as the reducing power involved in energy metabolism; secondly, as the cofactors or substrates of various enzymes participated in metabolic regulation or protein modification; thirdly, derivatives of these pyridine nucleotides act as messenger molecules involving in multiple signaling pathways to regulate the growth and development of organisms (Kirsch and De Groot, 2001; Moller, 2001; Berger et al., 2004; Ying, 2008).

Interestingly, the roles of NAD(H) and NADP(H) in different subcellular compartments may be somewhat different. For instance, in cytosol, when glycolysis is coupled to oxidative phosphorylation, cytosol NADH is shuttled by glyceraldehyde-3-phosphate dehydrogenase (GAPDH) into the mitochondrial matrix for consumption by the respiratory chain (Rossignol et al., 2004; Kelly et al., 2018). In addition, the fatty acid elongation cycle includes two reduction steps catalyzed by beta keto-ACP reductase (FabG) and enoyl-ACP reductase (FabI), respectively, in which FabI can use either NADH or NADPH as cofactor while FabG only uses NADPH in *E. coli* (Bergler et al., 1996; Zhang H. et al., 2017; Li W. et al., 2018). NADPH also helps in cellular anti-oxidation through following three ways: participating in the synthesis of glutathione (GSH), which is essential for several antioxidant enzymes (Valentine and Paglia, 1980; Ying, 2008); binding to H_2_O_2_-disposing enzyme catalase to reactivates catalase (Kirkman and Gaetani, 1984); and as essential component in the thioredoxin system that is another important antioxidation system (Arner and Holmgren, 2000; Li B.B. et al., 2018). In addition, as mentioned above, the cytosol NADPH can be also used by NADPH oxidases for apoplastic ROS production. In mitochondria, NAD(H) participate in TCA cycle and NADH can be also conversed into NADPH by transhydrogenases for the NADPH-dependent H_2_O_2_ scavenging system. In chloroplasts, NAD(H) and NADP(H) not only function in redox balance of this organelle, but also provide the reducing power for carotenol synthesis and also chloroplast-located NADPH-dependent H_2_O_2_ scavenging system. In peroxisomes, NADPH is also used for fatty acid β-oxidation but also for the NADPH-dependent H_2_O_2_ scavenging system (Li B.B. et al., 2018).

Moreover, both NAD^+^ and NADP^+^ are precursors of messenger molecules involved in signal transduction. The N-glycosidic bond of NAD^+^ is cleaved by NAD-dependent enzymes to leave an ADP-ribose host and a nicotinamide ring, then these decomposition products can take part in NAD-dependent signaling (Dolle et al., 2013). NAD-dependent enzymes can be divided into four main groups including PARP family [poly(ADP-ribose) polymerases], Sir2 family (NAD-dependent histone deacetylases), ARTs family [mono(ADP-ribosyl)transferases] and ARCs/cARHs family (ADP-ribosyl cyclases/cyclic ADP-ribose hydrolases) that play critical roles in DNA repair, gene expression, genomic stability, cell cycle, cell death, aging, carcinogenesis, calcium homeostasis and immune response by consuming NAD (Virag and Szabo, 2002; Denu, 2005; Grubisha et al., 2005; Sauve et al., 2006; Schreiber et al., 2006; Ying, 2006, 2008; Vyas et al., 2013). These enzymes can produce NAD derivatives, like cARDP which plays an important regulatory role in the maintenance of intracellular calcium homeostasis as well as NAADP, a well-known derivative of NADP^+^ (Patel et al., 2001). Furthermore, NAD^+^ also serves as substrate for protein modification including protein deacetylation, as well as mono- and poly-ADP-ribosylation (Berger et al., 2004). All these results showed that both the NAD(H) and NADP(H) have specific roles in varied aspects of cell growth and development regulation.

## NADK Regulates the Balance Between NADP(H) and NAD(H)

Although NAD(H) and NADP(H) have their unique biological functions, the proper NADP(H)/NAD(H) ratio in different cell compartments is crucial for both the plant normal growth regulation and stress tolerance (Takahashi et al., 2009). For example, in the chloroplast, it is necessary to maintain a relatively high ratio of NADP^+^/NAD^+^ for photosynthetic electron transport, and in the cytoplasm also needs an appropriate NADP(H)/NAD(H) ratio to ensure normal course of biological processes such as signal transduction and energy metabolism (Takahashi et al., 2006; Hashida et al., 2018). In addition, some studies have shown that the balance of NADP^+^/NAD^+^ is closely related to cell growth and metabolic processes (Hashida et al., 2009; Takahashi et al., 2009). Notably, redox homeostasis [NAD(P)H/NAD(P)^+^] associated with the NADP(H)/NAD(H) ratio is equally important. As the “Central modulator” of plants, redox homeostasis can maintain normal cell development and environmental adaptability (Paciolla et al., 2016). In fact, an earlier study has shown that the ratios of NADH/NAD^+^ and NADPH/NADP^+^ can be enhanced when the plants were subjected to drought stress conditions (Chen et al., 2004).

### NADK Is a Key Regulator of NADP(H)/NAD(H) Ratio

As reported previously, NADK is a highly conserved enzyme in NAD_kinase domain, functioning as *de novo* synthesis of NADP from the substrates NAD and ATP, which further plays an important role to balance the homeostasis between NAD(H) and NADP(H) pools (McGuinness and Butler, 1985; Kawai et al., 2001a,b; Lerner et al., 2001; Li B.B. et al., 2018). When subjected to different environmental conditions or adversities, the organism needs to quickly adjust the NADP(H)/NAD(H) ratio in the cell to cope against these adverse events, which requires the participation of NADK.

Different plant species have different numbers of NADKs, and these NADKs are distributed in different intracellular compartments (Li B.B. et al., 2018). In *Arabidopsis thaliana*, three NADK isoforms were identified. Among them, AtNADK1 and AtNADK3 are located in the cytoplasm and peroxisome, respectively, and are mainly involved in the supply of intracellular NADPH and against oxidative stress (Chai et al., 2006; Waller et al., 2010). While, AtNADK2 is located in chloroplast and plays a crucial role in NADP synthesis (Turner et al., 2004; Berrin et al., 2005; Chai et al., 2005, 2006). In wheat, total four NADKs were found, where TaNADK1 and TaNADK2 are located in the cytoplasm while TaNADK3 and TaNADK4 are localized in chloroplasts and peroxisomes, respectively (Wang et al., 2016). These findings suggested that plant NADKs regulate NADP(H)/NAD(H) ratio in different compartments of cells. However, how these NADKs accurately regulate the balance of NAD(H) and NADP(H) pool in their districts is largely unknown, yet.

Takahashi et al. (2006) found that the NADK activity in an *Arabidopsis NADK2* gene knockout mutant *nadk2* is decreased to 25% compared with the wild-type plants, causing a modification in the NADP^+^/NAD^+^ ratio, which ultimately affected cell growth. A few years later, Takahashi and colleagues created the *NADK2*-overexpressing transgenic *Arabidopsis* plants to study the impacts of altering NADP^+^/NAD^+^ ratio on plant metabolism. They found that the NAD pool in the *NADK2*-overexpressors is lower while the NADP pool is higher, leading to a 1.5–1.7 time higher NADP^+^/NAD^+^ ratio in the transgenic plants than the wild type. In contrast, the ratios of NADP^+^/NAD^+^ in wild type and *nadk2* mutant are 0.41 and 0.11, respectively (Takahashi et al., 2009). At the same time, the concentrations of several metabolites involved in the Calvin cycle, glutamine, glutamate as well as some other amino acids are higher in the overexpressors. These results indicated that changes of the NADP^+^/NAD^+^ ratio in overexpressing lines of *NADK2* directly or indirectly stimulate carbon and nitrogen assimilation in *Arabidopsis* (Takahashi et al., 2009), suggesting the vital roles of NADKs in the NADP(H)/NAD(H) balance of plants.

### Allosteric Regulation Mode of NADK to the Balance of NADP(H)/NAD(H)

So how does NADK regulate the ratio of NADP(H)/NAD(H)? The answer to this question may be related to the allosteric regulation mode of the enzyme. NADK catalyzes the synthesis of NADP by using ATP to phosphorylate NAD, but an excess of NADP and NAD analogs can inhibit the activity of NADK, which is called the allosteric regulation mode of NADK (Li B.B. et al., 2018). This allosteric regulation of NADK is ubiquitous in the animal and plant world.

Previously, it was noticed that the activity of NADK in human is regulated by NADPH and NADH (Ohashi et al., 2011). By purifying human NADK and detecting its enzymatic activity under different substrate conditions, the activity of NADK exhibited a competitive inhibition by NADPH and NADH with the Ki value for the two substrate being 0.13 and 0.34 mM, respectively. The same phenomenon was also reported in pigeons that NADK’s activity is competitively inhibited by NADPH and NADH (Apps, 1968). At the same time, NADH was found to be a very potent competitive inhibitor of NAD phosphorylation by NADK in spinach (Yamamoto, 1966). Thereafter, considerable evidence showed that there is also an allosteric regulation pattern of NADK in microorganisms (Zerez et al., 1986, 1987; Garavaglia et al., 2003; Ochiai et al., 2004; Paoletti et al., 2016). The inhibitor of NADK during aerobic growth in *Salmonella enterica* is NADPH, while NADH could inhibit NADK’s activity during the anaerobic growth. When no inhibitor is present, NADK exists as an equilibrium mixture of dimers and tetramers (KD = 1.0 ± 0.8 mM), while in the presence of NADPH, all NADKs are converted to tetrameric forms (Grose et al., 2006).

## Ca^2+^/CaM Complex Regulates NADK Activity

Many studies have shown that NADK-mediated NADP(H)/NAD(H) balance plays a key role in plant resistance to various stresses such as cold, salt, and drought (Zagdanska, 1990; Delumeau et al., 2000; Ruiz et al., 2002; Noctor et al., 2006). NADK also participates in the response of both plants and animals to oxidative stress by regulating the ratio of NADP(H)/NAD(H) (Harding et al., 1997; Gray et al., 2012). In the response of NADK to these environmental stresses, CaM plays a crucial role for the regulation of NADK activity (Cormier et al., 1981; Delumeau et al., 2000; Ruiz et al., 2002; Zeng et al., 2015).

### Ca^2+^ Signaling and CaMs

Ca^2+^ (Calcium ions) can serve as a versatile well-known cellular second messenger in signal transduction during various developmental processes and in response to environmental stresses. During the past few decades, researches about Ca^2+^ signaling pathways are very extensive but some of these pathways still worth exploring. Although higher concentrations of Ca^2+^ are harmful to plants and animals, it is essential to maintain a proper Ca^2+^ concentration in the cytoplasm for these organisms (Kudla et al., 2010). There are numerous review articles about Ca^2+^ signals which have made a commendable summary of this messenger in different focuses (Qudeimat and Frank, 2009; Dodd et al., 2010; Kudla et al., 2010; Batistic and Kudla, 2012).

After sensing development-related signals and stimulation by biotic/abiotic stresses, the organisms stimulate the opening of Ca^2+^ channels and pumps located on plasma membrane and/or endomembrane systems to further induce some rapid and transient changes in intracellular Ca^2+^ concentration (Ca^2+^ transients), ultimately producing a stimulating specific physiological response (DeFalco et al., 2009; Dodd et al., 2010; Batistic and Kudla, 2012). The temporal and spatial distribution changes in cellular Ca^2+^ concentrations caused by specific stimuli are defined as “Ca^2+^ signatures” (Qudeimat and Frank, 2009; Kudla et al., 2010; Batistic and Kudla, 2012). These Ca^2+^ signatures are detected, decoded and transmitted to affect downstream protein-protein interactions, phosphorylation cascades, or transcriptional responses by a diversity of Ca^2+^ sensors which further bind Ca^2+^ using the evolutionarily conserved EF-hand motif. These EF-hand Ca^2+^ sensors are divided into three major categories in plants: CaMs (Calmodulins) and CMLs (Calmodulin-like proteins); CDPKs/CPKs (Ca^2+^-dependent protein kinases); and CBLs (Calmodulin B-like proteins) and CIPKs (CBL interacting protein kinases). All of these Ca^2+^ sensors have been widely elucidated functioning in various biological processes (DeFalco et al., 2009; Batistic and Kudla, 2012; Xu et al., 2015).

As one kind of the major Ca^2+^ sensors, CaMs are essential for cells to interpreting encrypted Ca^2+^ signals. There are multiple cDNA sequences encoding CaMs which have been isolated and characterized from various organisms. In the 1980s, CaM was discovered from vertebrates including human (Sasagawa et al., 1982), eel electroplax (Lagace et al., 1983), chicken (Putkey et al., 1983), *Xenopus laevis* (Chien and Dawid, 1984), sea urchin (Floyd et al., 1986), and rat (Nojima and Sokabe, 1987). Until 1989, the first plant CaM cDNAs were successfully cloned in barley (*Hordeum vulgare L.*) using a plant-specific degenerate CaM oligonucleotide probe (Ling and Zielinski, 1989). Thereafter, more plant CaMs were identified in different species. There are nine CaM isoforms in *Arabidopsis thaliana* (Ling et al., 1991; Perera and Zielinski, 1992; Gawienowski et al., 1993; Zielinski, 2002) and three types of CaMs, which are encoded by 13 genes, in tobacco (*Nicotiana tabacum*) (Yamakawa et al., 2001). Meanwhile, depending on the number of exons, at least two distinct isoforms of CaMs encoded by eight genes were identified in potato (Takezawa et al., 1995). Multiple CaM members indicated they may have different physiological functions in plants. In addition, by interacting and regulating a large number of target proteins, including protein kinases, protein phosphatases, transcription factors and metabolic enzymes, Ca^2+^/CaM complex (Ca^2+^-loaded CaM) directly or indirectly participate in the regulation of plant responses to external environmental stresses (Zeng et al., 2015). But how do these diverse Ca^2+^-loaded CaMs specifically recognize and bind to their target proteins for the modulation and coordination of cell-specific biological processes to accurately transmitting intracellular calcium signals, is still under investigation.

### CaM Activates NADK

According to related research reports, NADK is the first enzyme identified in peas to be activated by Ca^2+^-dependent CaM and is actually used as a tool to examine CaMs (Muto and Miyachi, 1977; Anderson and Cormier, 1978; Stinemetz et al., 1987). Further research indicated that this activation mechanism is ubiquitous in almost all of the life kingdoms.

It was shown that the activation of NADK in higher plants is completely dependent upon the Ca^2+^-binding formation of a Ca^2+^-loaded CaM complex (Jarrett et al., 1980). By treatment with EGTA (a Ca^2+^ chelating agent) and trifluoperazine (an inhibitor of CaM), the enzyme activity of NADK was inhibited. Later, Epel et al. (1981) found that the NADK activity of sea urchin eggs after fertilization is regulated by Ca^2+^ and CaM *in vitro*. Then, two extremely divergent CaM isoforms were identified in soybean; the highly conserved SCaM-1 is a NADK-activated isoform while SCaM-4 is a NADK-inhibited isoform (Walton et al., 2017). In tobacco, there are three types of CaM isoforms with different suitable Ca^2+^ concentration which have disparate abilities to activate target enzymes and the type I can effectively activate NADK in stimuli-induced conditions (Karita et al., 2004). At the same year, Turner et al. (2004) cloned and characterized two NADK isoforms from *Arabidopsis thaliana*. By using recombinant glutathione-*S*-transferase fusion proteins and CaM-affinity chromatography and immunoblot analyses, they identified that AtNADK2 is a CaM-binding protein. However, the molecular interactions between CaMs and NADKs have not been examined yet for a long time.

**FIGURE 1:**
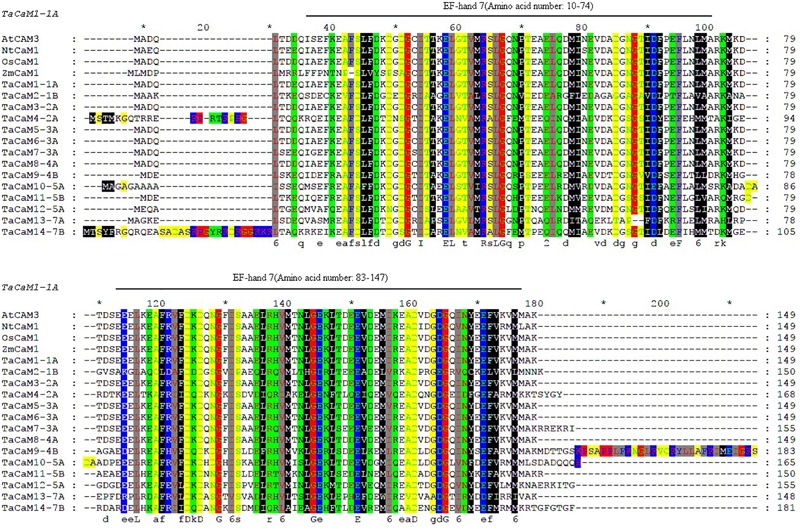
Sequence comparison of CaMs between wheat and other plant species generated with Clustalx and Gene Doc software. Gene ID for the sequences are as follows: AtCaM3 (824847), NtCaM1 (107763754), OsCaM1 (4332664), the transcript ID of ZmCaM1 is Zm00001d038545_T001, and the transcript ID of TaCaMs are shown in Table 1. Boxes of different colors represent amino acids of different physicochemical properties. Black lines above sequences denote EF-hand motifs of TaCaM1 analyzed in Pfam (http://pfam.xfam.org/). ^∗^Indicating the position of each 10 AAs in the full length AA sequence.

**FIGURE 2:**
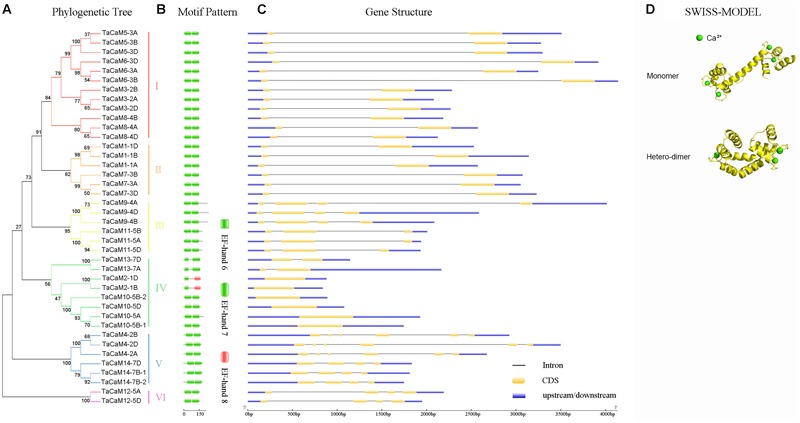
The phylogenetic relationship, conversed motifs, gene structure, and 3D protein structure analysis of *TaCaM* gene family. **(A)** Phylogenetic relationship of 40 TaCaM family members. The tree was constructed with MEGA7 using the maximum-likelihood method with the full-length *TaCaM* CDS sequences (from start codon to stop codon). Numbers above the nodes represent bootstrap values from 1,000 replications. According to the distance in the phylogenetic tree, we divided the 40 CaMs into six subfamilies (I–VI). **(B)** Domain organization of TaCaMs. Results from Pfam database (http://pfam.xfam.org/) show that all TaCaMs possess two EF-hand motifs at their C- and N-termini, respectively. **(C)** The exon/intron arrangement of *TaCaM* family genes. Analyzed in Gene Structure Display Server 2.0 (http://gsds.cbi.pku.edu.cn/). Black thin lines indicate introns, yellow areas indicate exons, and blue areas represent the untranslated region (3′ and 5′ UTR). **(D)** The typical structure models of TaCaM. The structure models were constructed using a homology modeling method with the full-length amino acid sequences of TaCaM3-2A. The EF-hand is a helix-loop-helix structure that usually binds two Ca^2+^. And two EF-hands connected by a long flexible helix. In addition, two CaM molecules can form a dimer.

**FIGURE 3:**
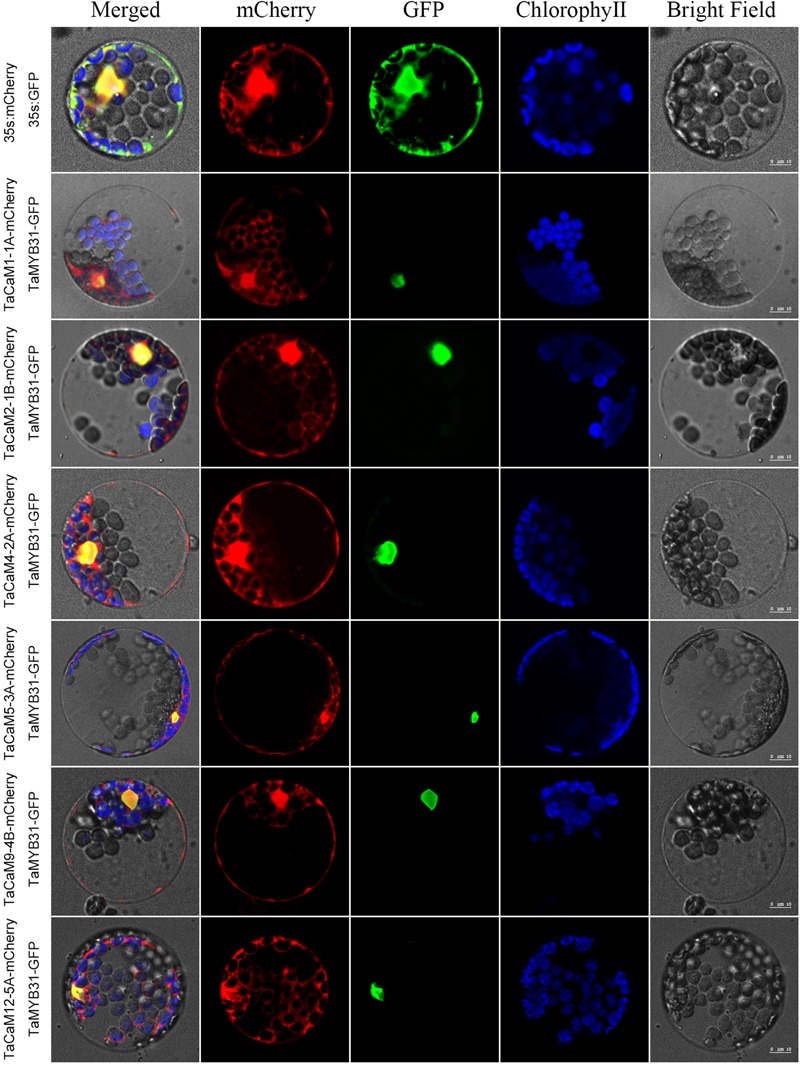
Subcellular localization of TaCaMs-mCherry fusion proteins in *Arabidopsis* protoplasts. The subcellular localization of the TaCaMs was analyzed in *Arabidopsis* mesophyll protoplasts according to the method of Yoo et al. (2007). Briefly, the plasmid constructs of 35S:GFP and 35S:mCherry, and 35S:TaMYB31-GFP and 35S:TaCaMs-mCherry were co-transformed into protoplasts prepared from *Arabidopsis* seedlings, and expression of the introduced genes was viewed after incubated 14 h by confocal microscopy (A1R, Nikon, Tokyo, Japan). For co-transformation, 15 μl plasmid DNA (around 15 μg) of each construct were added into a 2 mL microfuge tube and then 200 μl protoplasts were added. After mixed well gently, the protoplasts with plasmid were incubated overnight (14 h) with a gentle swirling motion at 40 rpm in the darkness. The GFP-fusion protein of TaMYB31 (KU674897.1), a nuclear transcription factor, was used as a marker of the nuclear localized protein. The mCherry signal indicates that the TaCaMs-mCherry proteins are located in the cytoplasm, nucleus, and plasma membranes.

In wheat, the latest theoretical research about the CaM family genes remains in the 1990s (Yang et al., 1996). With the continuous advancement of sequencing technology, new wheat CaM family members have emerged in the wheat databases. Here, we excavated 40 CaM genes from hexaploid bread wheat (*Triticum aestivum L.*) which encode 14 alleles CaM isoforms in the second generation wheat database (TGACv1^1^) by blasting with the known and typical CaM sequences of TaCaM1-1 (u48242) and AtCaM3 (AT3G56800), respectively. These CaM amino acid sequences share substantial identity with multiple plant CaM proteins from *Arabidopsis thaliana*, tobacco (*Nicotiana tabacum*), rice (*Oryza sativa Japonica*) and maize (*Zea mays*) (Figure 1). According to the arrangement of these genes on the chromosome, we named them to be TaCaM1-1A∼TaCaM14-7B-2 (Table 1). It is worth noting that 10 of these 40 CaMs have been demonstrated to have Ca^2+^-binding ability (Yang et al., 1996). Furthermore, the analyses for the exon/intron structure, functional domain and three-dimensional structure of these proteins with the advanced bioinformatic approaches including Gene Structure Display Server 2.0^2^, Pfam database^3^, and SWISS-MODEL-building models^4^, showed that, all of the TaCaMs have conserved a pair of EF-hands located at both the N- and C-terminus (Figure 2B–D). According to the phylogenetic tree of the TaCaMs and their gene structure and motif organization, we divided these TaCaM genes into six subfamilies (Figure 2A). By selecting the representative members in each of the six subfamilies, namely TaCaM1-1A, TaCaM2-1B, TaCaM4-2A, TaCaM5-3A, TaCaM9-4B, and TaCaM12-5A, the subcellular localization of these TaCaMs was analyzed and the results showed that all of the six selected TaCaMs are localized at the cytoplasm, nucleus, and plasma membranes (Figure 3), implying their multiple functions in wheat cells. This results are consistent with the previous reports that CaMs show different expression and localization and the majority of its targets are cytosolic or nuclear proteins (Perochon et al., 2011). CaM also binds to membrane-associated proteins (Perochon et al., 2011) and locates in plasma membranes (Collinge and Trewavas, 1989; Rodriguez-Concepcion et al., 1999). In rice, a CaM, OsCaM61 is membrane-associated but its unprenylated counterparts can be transported into nucleoplasm (Dong et al., 2002). Since two wheat NADKs, namely TaNADK1 and 2 are localized at the cytoplasm (Wang et al., 2016), we then checked the possible interaction relationships of these TaCaMs with the two TaNADKs by using a split luciferase system in tobacco leaves (firefly luciferase complementation assay). The results showed that TaCaM1/2/4/5/9 (TaCaM1-1A, TaCaM2-1B, TaCaM4-2A, TaCaM5-3A, TaCaM9-4B) could bind to TaNADK1/2, while TaCaM12-5A could not (data not shown), providing a preliminary theoretical basis for further studying the mechanism of TaCaMs-mediated activation of TaNADKs in wheat.

**Table 1:** *TaCaM* genes encoding CaM proteins along with their molecular details.

Gene name	Transcript ID	Number of predicted alternative transcript	Chromosome	Genome location	ORF (bp)	Length (Aa)	MW (kDa)	PI	Exon no.
TaCaM1-1A	TRIAE_CS42_1AL_TGACv1_001781_AA0035110	1	1A	499786745–499784449	450	149	16.84	3.89	2
TaCaM1-1B	TRIAE_CS42_1BL_TGACv1_031515_AA0115380	0	1B	543500203–543497067	450	149	16.84	3.89	2
TaCaM1-1D	TRIAE_CS42_U_TGACv1_641303_AA2091250	0	1D	404140097–404137578	450	149	16.84	3.89	2
TaCaM2-1B	TRIAE_CS42_1BL_TGACv1_031515_AA0115390	0	1B	543496743–543495902	453	150	15.93	4.21	1
TaCaM2-1D	TRIAE_CS42_U_TGACv1_641303_AA2091240	0	1D	404137527–404136649	453	150	15.83	4.23	1
TaCaM3-2A	TRIAE_CS42_2AS_TGACv1_112492_AA0339230	1	2A	51582187–51584265	450	149	16.83	3.88	2
TaCaM3-2B	TRIAE_CS42_2BS_TGACv1_148148_AA0490920	1	2B	77168807–77171085	450	149	16.83	3.88	2
TaCaM3-2D	TRIAE_CS42_2DS_TGACv1_178522_AA0597010	0	2D	49875788–49878051	450	149	16.83	3.88	2
TaCaM4-2A	TRIAE_CS42_2AS_TGACv1_113309_AA0354280	0	2A	119013443–119010775	507	168	19.34	4.43	6
TaCaM4-2B	TRIAE_CS42_2BS_TGACv1_146510_AA0467200	0	2B	167862799–167859875	507	168	19.34	4.43	6
TaCaM4-2D	TRIAE_CS42_2DS_TGACv1_178764_AA0600670	0	2D	117394444–117390949	507	168	19.34	4.43	6
TaCaM5-3A	TRIAE_CS42_3AS_TGACv1_211427_AA0690060	2	3A	246044717–246041212	450	149	16.8	3.88	2
TaCaM5-3B	TRIAE_CS42_3B_TGACv1_223954_AA0790090	0	3B	266565309–266567265	450	149	16.8	3.88	2
TaCaM5-3D	TRIAE_CS42_3DS_TGACv1_271624_AA0904180	0	3D	185789908–185793199	450	149	16.8	3.88	2
TaCaM6-3A	TRIAE_CS42_3AS_TGACv1_211977_AA0696260	0	3A	215482341–215479893	450	149	16.83	3.88	2
TaCaM6-3B	TRIAE_CS42_3B_TGACv1_224830_AA0802120	1	3B	254780109–254775974	450	149	16.83	3.88	2
TaCaM6-3D	TRIAE_CS42_3DS_TGACv1_272530_AA0921860	0	3D	175408560–175404646	450	149	16.83	3.88	2
TaCaM7-3A	TRIAE_CS42_3AL_TGACv1_195082_AA0644480	1	3A	581271638–581274686	450	149	16.79	4.10	2
TaCaM7-3B	TRIAE_CS42_3B_TGACv1_224708_AA0800320	1	3B	578399017–578402086	447	148	16.67	4.11	2
TaCaM7-3D	TRIAE_CS42_3DL_TGACv1_249883_AA0858060	1	3D	441144853–441148082	450	149	16.79	4.10	2
TaCaM8-4A	TRIAE_CS42_4AS_TGACv1_306509_AA1009430	0	4A	163816833–163819410	450	149	16.81	3.88	2
TaCaM8-4B	TRIAE_CS42_4BL_TGACv1_320285_AA1034000	0	4B	390279370–390281551	450	149	16.83	3.88	2
TaCaM8-4D	TRIAE_CS42_4DL_TGACv1_342497_AA1115220	0	4D	312802093–312799969	450	149	16.81	3.88	2
TaCaM9-4A	TRIAE_CS42_4AL_TGACv1_289299_AA0968690	0	4A	466579288–466576899	687	228	25.86	4.54	4
TaCaM9-4B	TRIAE_CS42_4BS_TGACv1_327875_AA1077330	0	4B	171614995–171612913	696	231	26.03	4.64	4
TaCaM9-4D	TRIAE_CS42_4DS_TGACv1_362084_AA1176580	0	4D	110110459–110113042	726	241	27.18	4.76	4
TaCaM10-5A	TRIAE_CS42_5AS_TGACv1_393767_AA1275870	0	5A	54463490–54464541	498	165	18.14	3.90	1
TaCaM10-5B-1	TRIAE_CS42_5BS_TGACv1_423346_AA1374830	0	5B	66054115–66052365	507	168	18.56	3.90	1
TaCaM10-5B-2	TRIAE_CS42_5BS_TGACv1_423346_AA1374840	0	5B	66036793–66037681	492	163	18.02	3.84	1
TaCaM10-5D	TRIAE_CS42_5DS_TGACv1_457623_AA1488360	0	5D	64134020–64135098	507	168	18.52	3.90	1
TaCaM11-5A	TRIAE_CS42_5AL_TGACv1_378470_AA1253490	0	5A	311946816–311944877	543	180	19.96	4.10	3
TaCaM11-5B	TRIAE_CS42_5BL_TGACv1_406602_AA1347650	0	5B	256917263–256915255	543	180	20.06	4.18	3
TaCaM11-5D	TRIAE_CS42_5DL_TGACv1_434188_AA1431320	0	5D	241954475–241952547	543	180	19.84	4.15	3
TaCaM12-5A	TRIAE_CS42_5AL_TGACv1_375888_AA1228470	0	5A	580799769–580801950	468	155	17.46	3.81	4
TaCaM12-5D	TRIAE_CS42_5DL_TGACv1_434791_AA1441690	0	5D	460647918–460649869	468	155	17.47	3.81	4
TaCaM13-7A	TRIAE_CS42_7AL_TGACv1_557585_AA1783610	2	7A	450962273–450964141	447	148	16.73	4.78	2
TaCaM13-7D	TRIAE_CS42_U_TGACv1_644336_AA2138900	0	7D	396981643–396982292	447	148	16.76	4.78	2
TaCaM14-7B-1	TRIAE_CS42_7BL_TGACv1_577684_AA1881050	0	7B	680163247–680161440	546	181	20.63	4.52	4
TaCaM14-7B-2	TRIAE_CS42_7BL_TGACv1_577911_AA1885910	0	7B	680163327–680161583	546	181	20.63	4.52	4
TaCaM14-7D	TRIAE_CS42_7DL_TGACv1_603770_AA1988940	0	7D	600968822–600966986	546	181	20.66	4.48	4

## The Mechanism of CaM-Mediated Activation of NADK

### Structural Properties of CaMs and NADKs

Calmodulins are a class of very small and heat-stable protein that consists of approximately 148–200 amino acids that transmit Ca^2+^ signatures in almost all of eukaryotes (Anderson and Cormier, 1978). Every conservative CaM possesses a pair of EF-hand domain at its C- and N-termini connected by a long flexible helix. The EF-hand domain is a helix-loop-helix structure that usually binds two Ca^2+^ (Snedden and Fromm, 2001). In addition to EF-hands, there are no other function known domains existing in the CaMs, so the small acidic proteins generally do not have enzymatic or biochemical functions except for binding Ca^2+^ (Zeng et al., 2015). CaM itself has no catalytic activity, but it can activate many target proteins as long as it combined with Ca^2+^ to involve in a variety of cellular processes. Most of CaMs work in a monomeric form, however, recent studies have shown that CaMs may also exist as X-shaped dimer (Figure 2D) (Larsson et al., 2005; Zhang et al., 2008).

NAD kinases is a well-studied CaM effector protein and is expressed almost throughout the growth of plants, suggesting that NADK plays an integral role in plant growth and development (Li B.B. et al., 2018). A typical tertiary structure of NADK consists of an N-terminal domain (NTD) and a C-terminal domain (CTD), and the catalytic site is located in the deep crack between NTD and CTD (Li B.B. et al., 2018). The most basic function of NADKs is to phosphorylate NAD at the 2′ position of the adenosine ribose using phosphoryl donor ATP to synthesize NADP and balances the cellular redox state in living organisms (Magni et al., 2006). The structure and function of NADK family members are relatively conservative, but they have considerable different motifs. According to our previous research, we divided 74 NADK genes from 24 plant species into four subfamilies (I–IV) on the basis of a rooted maximum likelihood phylogenetic tree (Li et al., 2014). Further studies showed that there are large differences in motif organization between different NADK subfamilies (Li et al., 2014). Although all identified NADKs contain a typical NAD_kinase domain at the C-terminus, the proteins which belong to subfamily II carry an additional N-terminal catalytic domain and subfamily III NADKs own two NAD_kinase domains (Li et al., 2014). Depending on whether it can be activated by CaM, NADK is also divided into CaM-dependent and -independent isoforms (Turner et al., 2004; Li B.B. et al., 2018).

### CaM Binding Domain (CaMBD)-Based Regulation

CaM can interact with its target kinases through a hydrophobic and electrostatic manner by a short α-helix called CaM-binding domain (CaMBD), a stretch of approximately 20 amino acid residues with a positive charge (Snedden and Fromm, 2001; Hoeflich and Ikura, 2002; Zeng et al., 2015). Although most CaMBD peptides share a common conserved secondary structure, their primary sequences are diverse (Snedden and Fromm, 2001; Zeng et al., 2015). The CaM-dependent NADKs were reported to contain this domain. Turner et al. (2004) cloned a NADK from *Arabidopsis thaliana* named AtNADK2 and found that the N-terminal extension of the protein contains a CaMBD, which could bind to CaM-agarose and is necessary for the CaM combination with NADK. In addition to *Arabidopsis*, NADKs containing CaM-binding domain are also present in other plants, such as rice (AK065215), lettuce (BQ998926), soybean (AW620785), wheat (BE402274), and sorghum (BE364471) (Turner et al., 2004).

However, we found that wheat TaNADK1 and 2 do not contain the CaMBD motif by a sequence alignment analysis using a comprehensive Calmodulin Target Database maintained by Ikura Labs at the Toronto University^5^ (Yap et al., 2000; Snedden and Fromm, 2001). Only a potential CaM binding site can be identified in TaNADK1 and 2 but needs further experimental verification.

### CaM 115-Lys Methylation-Involved Regulation

Beside the CaMBD present on NADK, CaM 115-Lys methylation also affects the activation effect of CaM to NADK. Calmodulin *N*-methyltransferase (CaM KMT) is an evolutionarily conserved enzyme responsible for the formation of trimethyllysine in CaM (Magnani et al., 2010; Banerjee et al., 2013). Magnani et al. (2012) demonstrated that the methylation affects the conformational dynamics of CaM upon binding of Ca^2+^, however, the function of the conserved CaM trimethylation is still less to be understood. A limited number of studies showed that most of the CaMs isolated from plant tissues are mostly methylated post-translationally at 115-lysine and this methylation state significantly affects their ability to activate plant NADKs (Roberts et al., 1986; Zielinski, 1998; Banerjee et al., 2013).

Roberts and coworkers constructed two foreign CaMs (VU-1 and VU-3 calmodulins) transgenic tobacco plants both under the control of the cauliflower mosaic virus 35S promoter to study the effect of CaM methylation on NADK activity (Roberts et al., 1992). VU-1 CaM has a methylated lysine residue at position 115, while lysine at position 115 of VU-3 CaM is replaced by arginine which cannot be trimethylated and the remaining sequence is identical to VU-1 CaM. Their conclusions indicated that VU-3 CaM (Lys to Arg115) retains the same CaM activity as methylated CaM, but hyperactivates NADK compared with VU-1 CaM *in vitro*. Moreover, some other studies showed that this conserved 115-Lys methylation may also serve an important function of protecting CaMs from ubiquitination and degradation via the ubiquitin-dependent pathway *in vivo* (Gregori et al., 1987). These studies also explained the causes about the dysplasia of the line expressing the VU-3 CaM: reduced CaM methylation may perturb the sequence of events of DNA replication or chromosome partitioning in meiosis (Zielinski, 1998). Taken together, these results not only proved the sensitivity of plant NADKs to the methylated CaMs but also provide a precedent for a functional effect of CaM methylation which deserves more in-depth research in plants and animals.

### Ca^2+^/CaM Complex Dependent Regulation

As talked above, CaM acts as a molecular switch after binding with Ca^2+^, and thereby regulates many downstream target proteins by transmitting the calcium signals. In other words, most of the activation of downstream target proteins by CaMs is Ca^2+^-dependent. NADK is a well-studied example that activated by Ca^2+^/CaM complex. Although plentiful studies have shown that CaM-dependent NADK exists in plants and animals, little research has been reported on the molecular activation mechanism of NADKs by CaMs. At the same time, CaM-independent NADK isoforms also exist in the biological world. Hence, CaMs may regulate the enzyme activity of individual NADK through different pathways (Zielinski, 1998). Ca^2+^/CaM complex not only directly interacts with NADK and activates it, but also indirectly activates NADK through a Ca^2+^/CaM-mediated kinase cascade.

This hypothesis was confirmed in a subsequent experiment by Love et al. (2015). They identified two NADK isoforms from sea urchin (*Strongylocentrotus purpuratus*) and discovered that both the resultant NADKs exhibit kinase activity but differ in their N-terminal ∼90 amino acids. Furthermore, they found that there is a CaMBD at the N-terminus of SpNADK-2, whereas no CaMBD domain was observed in SpNADK-1 isoform through a functional domain analysis. Further experiments *in vitro* found that SpNADK-2 is a Ca^2+^/CaM-dependent NADK that can directly combine with CaM and be activated. Compared to SpNADK-2, both SpNADK-1 and human NADK N-terminals have phosphorylation sites, although there is no CaM binding site. In combination with previous studies on the phosphorylation of mouse NADK in the liver (Haas and Gygi, 2013), and the kinase CaMKII (CaM-dependent protein kinase II) which can phosphorylate NADK via activated by Ca^2+^/CaM (Griffith, 2004), Love et al. (2015) concluded that CaMKII may phosphorylate NADK in a Ca^2+^/CaM-dependent manner. In addition, they observed that only SpNADK-1 can be phosphorylated when incubating SpNADK-1 and SpNADK-2 were incubated with HeLa cells and [32P] ATP. Next, the phospho-sensitive site of the NADKs was determined: serine 18 of SpNADK-1 and serine 64 of human NADK, where human NADK has the same phosphorylation site as mouse NADK. The studies on the CaMKII-mediated phosphorylation of the NADK N-terminus not only revealed the molecular mechanisms of CaM-dependent regulation of NADKs in animals, but also provided a significative example for the complicated CaM regulatory network that has not been proved in plants.

### cADPR- and NAADP-Mediated Regulation

**FIGURE 4:**
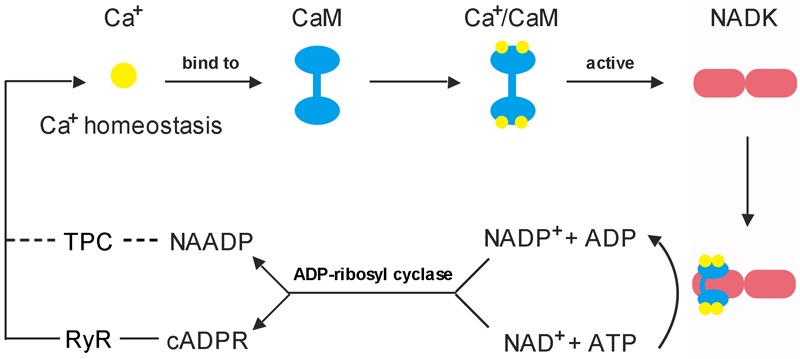
Feedback regulation mode of CaM on NADK activation. (1) Each CaM has the ability to bind four Ca^2+^ to form a Ca^2+^/CaM complex, and the Ca^2+^-loaded-CaM complex can interact with NADK to activate it (Love et al., 2015; Zeng et al., 2015). (2) NAD kinase is highly conserved in a function that responsible for the *de novo* synthesis of NADP from the substrates NAD and ATP, and plays an important role to balance the homeostasis between NAD pool and NADP pool (Chai et al., 2005; Li B.B. et al., 2018). (3) cADPR and NAADP are derived from NAD^+^ and NADP^+^, respectively, and play important roles in mobilizing intracellular Ca^2+^ stores and influencing Ca^2+^ homeostasis (Lee, 2012). These two Ca^2+^ second messengers can affect the binding of Ca^2+^ to CaM by causing intracellular Ca^2+^ transients, and finally, affect the binding of Ca^2+^/CaM complex to NADK.

Early in 1987, it was demonstrated that there are three independent Ca^2+^ release mechanisms induced by IP3 [Inositol trisphosphate (InsP3)], NAD and NADP (Clapper et al., 1987). However, subsequent studies showed that NAD and NADP-mediated Ca^2+^ release is due to their derivatives rather than themselves. cADPR and NAADP are two types of NAD and NADP derivatives which act as second messengers to mobilize intracellular Ca^2+^ stores through different pathways (Wu et al., 1997; Cancela et al., 1999). The property of cADPR and NAADP with Ca^2+^-mobilizing activity was firstly evaluated in sea urchin eggs (Lee and Aarhus, 1995). It is interesting to note that cADPR and NAADP are derived from NADP^+^ and NAD^+^, respectively, these two are essentially linked by NADKs. Hence, NADK may play a role in switching between these two signaling pathways (Figure 4).

cADPR is a cyclic molecule and its cyclization site situated N1-position of the adenine ring, which is linked to the anomeric carbon of the terminal ribose (Lee et al., 1994). When the cyclizing linkage was broken by enzymes or chemical factors, a conventional ADP-ribose is produced by adding a hydroxyl group to the terminal ribose and hydrogen to the 1-nitrogen of the adenine (Lee et al., 1994; Aarhus et al., 1995). It is a cyclic molecule and generates ADP-ribose after hydrolysis, so it is called cyclic ADP-ribose (cADPR) (Lee, 2001). Different with cADPR, NAADP is a linear molecule and its nicotinamide group replaced with a nicotinic acid group (Aarhus et al., 1995). Although the molecular structure of cADPR and NAADP is different, they are all synthesized by the same type of enzymes, which include ADP-ribosyl cyclase and its homolog, CD38 (Aarhus et al., 1995; Lee, 2012; Lin et al., 2017). Additionally, both the enzymes can catalyze the breakdown of cADPR into ADP-ribose, and CD38 can also hydrolyze NAADP to ADP-ribose phosphate (Kim et al., 1993; Graeff et al., 2006; Liu et al., 2009). Although both NAD^+^ and NADP^+^ can serve as substrates for ADP-ribosyl cyclase and CD38, the binding of the enzymes to the substrates appears to be selective. Evidence suggests that at acidic pH, the enzymes tend to select NADP^+^ to synthesize NAADP, while at neutral or alkaline pH, the enzymes primarily cyclize NAD^+^ to produce cADPR and convert NADP^+^ to another metabolite, cyclic ADP-ribose 2′-phosphate (Aarhus et al., 1995). This catalytic property also explains why NAADP can function in an acidic organelle such as lysosomes (Fang et al., 2018).

cADPR and NAADP exhibit many functions such as activation of gene expression and cell cycle regulation, but their most direct function is to mediate intracellular Ca^2+^ signaling. It was reported that cADPR has the ability to trigger cytosolic Ca^2+^ concentration elevation through ryanodine receptor (RyR) in the Endoplasmic reticulum (ER) by a Ca^2+^ cascade way (Meissner, 1994; Lokuta et al., 1998; Ogunbayo et al., 2011). In other words, cADPR-mediated Ca^2+^ release is induced by Ca^2+^ and known as the Ca^2+^-induced Ca^2+^ release (CICR) mechanism (Galione et al., 1991). This mechanism was first reported in sea urchin eggs, and it was later confirmed in cultured bullfrog sympathetic neurons and rat dorsal root ganglion (DRG) neurons (Currie et al., 1992; Hua et al., 1994). Evidence also suggested that CaM can act as a functional mediator of cADPR-triggered CICR, which means cADPR acts on RyRs via CaM (Lee et al., 1995). Further research found that cADPR-induced Ca^2+^ release is consisted of two phases, an initial rapid release phase and a subsequent slower release; CaM is activated by Ca^2+^ release during the initial phase and functions in the second phase (Tanaka and Tashjian, 1995). However, with the deepening of research, it was reported that the actual receptor for cADPR is FKBP12.6 (FK506 binding protein of 12.6 kDa) (Tang et al., 2002; Wang et al., 2004; Lee, 2012). By investigating the role and molecular mechanism of cADPR action on Ca^2+^ spark properties, Zheng et al. (2010) found that cADPR-induced Ca^2+^ release is mediated by FKBP12.6 proteins in mouse bladder smooth muscle. Furthermore, the latest study showed that GAPDH is one of the cADPR-binding proteins and is required for cADPR-mediated Ca^2+^ mobilization from ER via RyRs (Zhang K. et al., 2017).

However, relatively fewer studies on the cADPR-mediated Ca^2+^ release mechanisms have been done in plants. Nevertheless, there is still evidence showing that intracellular cADPR levels are increased after abscisic acid (ABA) stimulation in plants and cADPR can activate the expression of ABA-responsive related genes (Wu et al., 1997; Leckie et al., 1998). Allen et al. (1995) first discovered that cADPR is able to elicit Ca^2+^ release at the vacuolar membrane of beet storage root. In addition, it was reported that cADPR-induced Ca^2+^ release through RyRs can augment IP3-elicited Ca^2+^ mobilization, and a similar cADPR-gated Ca^2+^ release mechanism exists in plants (Berridge, 1993; Allen et al., 1995). A few years later, by investigating the guard cells after ABA treatment, it was confirmed that cADPR can indeed increase the intracellular Ca^2+^ concentration by mobilizing vacuolar Ca^2+^ storage (Leckie et al., 1998). Through a series of bioassays experiments in *Arabidopsis thaliana*, Wu et al. (1997) proposed that cADPR is responsible for initiating the cascade of Ca^2+^ increases and subsequent Ca^2+^-dependent phosphorylation and dephosphorylation during ABA signal transduction.

Differed from the activation of ER Ca^2+^ stores by cADPR, NAADP shows to trigger Ca^2+^ release from lysosomal Ca^2+^ stores (Churchill et al., 2002; Yamasaki et al., 2004). There is ample evidence showing that NAADP-induced Ca^2+^ release mechanisms are triggered by cholecystokinin (CCK) and the receptor for NAADP is the two-pore channels (TPCs) (Cancela et al., 1999; Calcraft et al., 2009; Pitt et al., 2010; Tugba Durlu-Kandilci et al., 2010; Kelu et al., 2018). Interestingly, it was suggested that both InsP3 and cADPR may also transduce CCK-receptor signaling through different receptor subtypes. In other words, CCK not only triggers NAADP-mediated calcium release but also transmits calcium signals through InsP3 and cADPR (Petersen et al., 1994; Cancela et al., 1998). In addition, an experimental data showed that cellular NAADP levels are increased immediately after CCK addition; whereas, although cADPR levels are also increased by CCK, the time course is much slower than NAADP (Yamasaki et al., 2005). Combined with these results, it seems that the Ca^2+^ storage in the ER and lysosomes appears to be somewhat related. Alternatively, the Ca^2+^ release from the lysosomal stores can activate further release from the ER stores via the CICR mechanism. This means that NAADP can act as a trigger, whose Ca^2+^ signal is then amplified through the Ca^2+^ release regulated by cADPR (Cancela et al., 1999; Lee, 2012).

Compared to animals, NAADP-induced Ca^2+^ release mechanisms are slightly different in plants. The NAADP-mediated Ca^2+^ store remains controversial. Previous studies showed that both cADPR- and InsP3-elicited Ca^2+^ can release at the vacuolar membrane and the vacuole is a major Ca^2+^ store in plants (Allen et al., 1995). However, after purified the vacuolar vesicles from red beet, it was found that the NAADP-sensitive Ca^2+^ pool does not reside at the vacuolar membrane; the microsomal vesicles might respond to NAADP (Navazio et al., 2000). Analysis with the sucrose gradient-separated cauliflower microsomes revealed that the NAADP-sensitive Ca^2+^ pool is derived from the ER, and furthermore, the NAADP-gated Ca^2+^ release is incapable to trigger CICR mechanism (Navazio et al., 2000).

## Conclusion and Future Directions

The Ca^2+^/CaM complex activates NADK, which can phosphorylate NAD to NADP and balances the ratio of NADP(H)/NAD(H), while the derivatives of NAD^+^ and NADP^+^, cADPR and NAADP, play a key role in regulating Ca^2+^ homeostasis *in vivo*. Such feedback regulation mechanism is necessary for maintaining the dynamic balance of various small molecules in cells, and ensuring the normal operation of life activities. More importantly, advanced research on animals also provides the necessary reference value for the study of this mechanism in plants.

However, a number of questions related to this feedback regulation mechanism in plants still remain to be addressed. For example in wheat: first, although there is an interaction between TaCaMs and TaNADK1/2, do TaCaMs have an activation effect on the TaNADKs? Second, what is the key site that affects the interaction between the TaCaMs and TaNADKs, including the determination of specific amino acid sites on TaCaMs and the CaM-binding site on TaNADKs. Third, the interaction mode between the TaNADKs and TaCaMs is the same, that is, they all interact with TaCaMs of the same subfamily, then are there any differences in their regulatory mechanisms? The revelation of these issues is significant to fully understand the regulation mechanism of CaMs to NADK, as well as to improve the stress tolerance of plants by using this regulation mechanism.

## Data Availability

We have cloned 17 TaCaM family genes and submitted these nucleotide sequences data to GenBank. Accession numbers of the partial TaCaM family genes we discussed in this manuscript are as follows: TaCaM1-1A (MK493479), TaCaM2-1B (MK493481), TaCaM4-2A (MK493484), TaCaM5-3A (MK493486), TaCaM9-4B (MK493490), and TaCaM12-5A (MK493493).

## Author Contributions

K-MC provided funding and proposed concept. LT wrote the manuscript. LT and B-BL contributed to experimental data. X-MN and P-PZ analyzed the data. C-HH, LZ, W-TL, W-QL, and K-MC revised the manuscript.

## Conflict of Interest Statement

The authors declare that the research was conducted in the absence of any commercial or financial relationships that could be construed as a potential conflict of interest.

## Footnotes

1 http://oct2017-plants.ensembl.org/index.html

2 http://gsds.cbi.pku.edu.cn/

3 http://pfam.xfam.org/

4 https://swissmodel.expasy.org/interactive

5 http://calcium.uhnres.utoronto.ca/ctdb/flash.htm
